# Automastoidectomy

**DOI:** 10.1016/S1808-8694(15)30982-4

**Published:** 2015-10-19

**Authors:** João Alcides Miranda, Fábio Akira Suzuki, Marcello Henrique de Carvalho Borges, Andre Luis Sartini

**Affiliations:** a Resident of the Otorhinolaryngology Unit of the Sao Francisco University Hospital, Bragança Paulista, SP; b Doctor in Otorhinolaryngology graduated at UNIFESP - EPM, Head of the Otorhinolaryngology Unit of the Sao Francisco University Hospital, Bragança Paulista, SP, Vice-coordinator of the post-graduate course in Otorhinolaryngology of the Servidor Publico Estadual Hospital (HSPE); c Resident of the Otorhinolaryngology Unit of the Sao Francisco University Hospital, Bragança Paulista, SP; d Mestrando em Otorrinolaringologia pelo Hospital do Servidor Público Estadual, Médico Assistente do Serviço de Otorrinolaringologia do Hospital Universitário São Francisco, Bragança Paulista. This study was done in the Sao Francisco University Hospital, Bragança Paulista - SP

**Keywords:** automastoidectomy, cholesteatoma, chronic otitis media

## INTRODUCTION

Automastoidectomy is defined as extensive destruction of the middle ear cavity and the mastoid, appearing as an image reminiscent of a radical mastoidectomy cavity.1 This condition may arise as a complication of cholesteatomatous chronic otitis media2 or of keratosis obturans.3 Very little, however, has been described of this entity in literature.

## CASE REPORT

**Figure 1:**
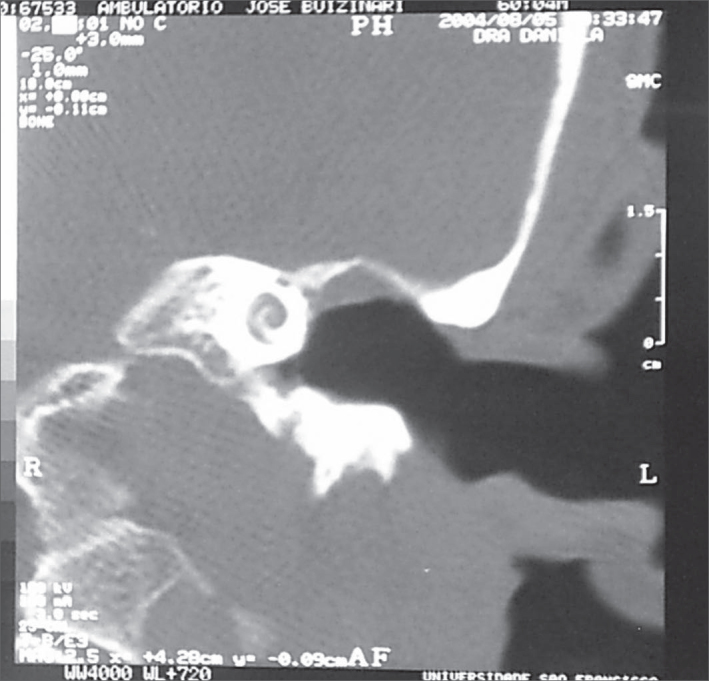
Computed tomography, coronal section, showing radical mastoidectomy cavity to the left.

A male 60 year old patient presented with left hypoacusia beginning 8 months ago. The patient complained of sporadic recurrent left otorrhea since infancy, the last episode occurring 18 months ago. Otoscopy revealed an intact opacified left tympanic membrane; the manubrium of the malleus was not seen. Audiometry showed left severe mixed hearing loss and moderate right neurosensorial hearing loss. Computed tomography of the ear disclosed a wide left tympanic cavity and absence of the ossicular chain (Figure 1). The diagnosis was left automastoidectomy. An exploratory tympanotomy was discussed but the patient refused surgical treatment.

## DISCUSSION

Automastoidectomy is a rare sequela of diseases that affect the external and middle ear. Hawke and Shanker3 report a case of automastoidectomy resulting from keratosis obturans. This report describes a patient with left automastoidectomy and a history suggesting chronic non-cholesteatomatous otitis media. Based on the patient's history and the image diagnosis, this appears to be a situation in which automastoidectomy is unrelated to a cholesteatoma or simple chronic otitis media that progressed following closure of a possible tympanic perforation. These conditions would not convincingly explain the bone destruction found in this case. Could this possibly be a case of cholesteatomatous chronic otitis media that hypothetically resolved spontaneously, leaving an automastoidectomy as a sequela? According to Hungria,4 this might be possible, however no similar report has been published in medical literature.

## CONCLUSION

Automastoidectomy is described as a rare complication of external and middle ear diseases; there has been no publication associating this condition with chronic otitis media other than the cholesteatomatous form of otitis media.
